# Trimethylamine-*N*-oxide (TMAO) and risk of incident cardiovascular events in the multi ethnic study of Atherosclerosis

**DOI:** 10.1038/s41598-025-05903-3

**Published:** 2025-07-02

**Authors:** Matthew J. Budoff, Marcia C. de Oliveira Otto, Xinmin S. Li, Yujin Lee, Meng Wang, Heidi T. M. Lai, Rozenn N. Lemaitre, Alan Pratt, Wai Hong Wilson Tang, Bruce M. Psaty, David S. Siscovick, Stanley L. Hazen, Dariush Mozaffarian

**Affiliations:** 1 Department of Medicine, Lundquist Institute at Harbor-UCLA, Torrance, CA USA; 2 Department of Epidemiology, The University of Texas Health Science Center at Houston (UTHealth) School of Public Health, Houston, TX USA; 3 Department of Cardiovascular and Metabolic Sciences, Lerner Research Institute, Cleveland, OH USA; 4 Center for Microbiome and Human Health, Lerner Research Institute, Cleveland, OH USA; 5 Department of Food and Nutrition, Myongji University, Yongin, Korea; 6 Food is Medicine Institute, Tufts University, Boston, MA USA; 7 Department of Primary Care and Public Health, Imperial College London, London, UK; 8 Cardiovascular Health Research Unit, Department of Medicine, University of Washington, Seattle, WA USA; 9 Department of Cardiovascular Medicine, Heart, Vascular and Thoracic Institute, Cleveland Clinic, Cleveland, OH USA; 10 Department of Epidemiology, University of Washington, Seattle, WA USA; 11 Department of Health Systems and Population Health, University of Washington, Seattle, WA USA; 12 The New York Academy of Medicine, New York City, NY USA

**Keywords:** Cardiovascular disease (CVD), Major adverse cardiac events (MACE), Myocardial infarction, Trimethylamine N-oxide (TMAO), Nutrition, Cardiology, Medical research, Risk factors

## Abstract

Trimethylamine-*N*-oxide (TMAO) is a gut microbiome-derived metabolite of choline, L-carnitine and lecithin, abundant in animal source foods. In experimental models, higher blood TMAO levels enhance atherosclerotic cardiovascular disease (ASCVD). However in humans, most prior studies have evaluated high risk or secondary prevention populations, and no studies have investigated relationships in a diverse, multi-ethnic population. We evaluated 6,767 US adults free of ASCVD at baseline in the community-based Multi-Ethnic Study of Atherosclerosis (MESA), including 38% identifying as White; 28%, as Black; 22%, as Hispanic; and 12%, as Chinese adults. Plasma TMAO was measured serially at baseline and 5-years, and its time-varying association with incident ASCVD determined using Cox proportional hazards. Multivariate analyses adjusted for time-varying demographics, lifestyle factors, medical history, lipid measures, antibiotic use and dietary habits. During median 11.3 years follow-up, 852 ASCVD events occurred. After multivariate adjustment, TMAO associated with higher risk of ASCVD in a dose-dependent fashion, with hazard ratios across quintiles of 1.02, 1.17, 1.23, and 1.33 (95% CI 1.02, 1.74), respectively, compared to the lowest quintile (P-trend = 0.01). Risk appeared possibly larger among Hispanic and Chinese adults; and among individuals with lower baseline renal function; although these interactions did not achieve statistical significance. Plasma concentrations of TMAO associated with higher risk of incident ASCVD in this multi-ethnic US cohort, supporting a need to test dietary and pharmacologic interventions targeting the diet-microbiome axis for potential cardiovascular risk prevention in diverse populations.

**Supplementary Information:**

The online version contains supplementary material available at 10.1038/s41598-025-05903-3.

## Condensed abstract

Trimethylamine-*N*-oxide (TMAO) is a gut microbiome-derived metabolite of nutrients more abundant in animal source foods. We evaluated 6,767 US adults free of ASCVD at baseline in the population-based Multi-Ethnic Study of Atherosclerosis. Plasma TMAO was measured at baseline, and its association with incident atherosclerotic cardiovascular events over 11.3 years. In multivariate models, TMAO was associated with higher risk of ASCVD, with HRs across quintiles of 1.02, 1.17, 1.23, and 1.33 (95% CI 1.02, 1.74), respectively, compared to the lowest quintile (P-trend = 0.01). Plasma TMAO was associated with higher risk of incident ASCVD in this multi-ethnic cohort of middle-aged U.S. adults.

Growing evidence supports an important role of gut microbiota in the development of atherosclerosis cardiovascular disease (ASCVD)^1–4^. Trimethylamine N-oxide (TMAO) is a gut microbiota-derived metabolite of dietary choline, L-carnitine, and phosphatidylcholine (lecithin)-rich animal foods (e.g., especially red meat and eggs; also fish and poultry). Based on experimental animal studies, combined with numerous clinical studies of high risk patients enriched with prevalent disease, elevated plasma TMAO may increase risk of ASCVD^5–10^. However, very few studies have examined the relationship of TMAO with onset of ASCVD in a well-characterized, community-based multi-ethnic cohort free of ASCVD at baseline^8^^9^.) This extensively phenotyped Multi-Ethnic Study of Atherosclerosis has advantages of it’s longitudinal nature, carefully adjudicated outcomes, multiple measures of TMAO, and racial/ethnic diversity. Understanding the epidemiology of the effect of TMAO on ASCVD is important, and elucidating these relationships is important to understand the generalizability of potential effects of TMAO across different race/ethnicity groups (as risk factor prevalence, social determinants of health and atherosclerosis severity all vary by race/ethnicity), and to address the potential for confounding and reverse causation in clinical samples of high risk patients with prevalent disease. In addition, TMAO levels are known to change over time due to variation in diet, the microbiome, and metabolism. Few studies have assessed ASCVD risk using baseline and 5-year follow-up measures of TMAO over time, rather than a single baseline measure.

To address these gaps, we investigated whether baseline and 5-year follow-up measures of plasma TMAO were associated with incident ASCVD risk in the MESA, a large prospective, community-based, multi-racial/ethnic cohort with extensive phenotyping. We hypothesized that higher levels of plasma TMAO would be associated with higher ASCVD risk.

## Methods

### Study design and recruitment

MESA^11^ is a longitudinal, population-based cohort study of incident ASCVD funded by the National Heart, Lung, and Blood Institute. A total of 6,814 men and women, aged 45–84 years and free of clinical cardiovascular disease at baseline, were recruited from six Field Centers in Baltimore, MD; Chicago, IL; Forsyth County, NC; Los Angeles, CA; New York, NY; and St. Paul, MN. Racial/ethnic groups enrolled included White, Black, Hispanic, and Chinese adults. Approximately 50% of the participants enrolled were female. The baseline visit took place between July 2000 and September 2002, with in-person study follow-up visits approximately every 2.5 years. The study was approved by Institutional Review Boards at each site and informed consent was obtained from all participants and/or their legal guardians. This study was approved by the Institutional Review Board at the Lundquist Institute at Harbor-UCLA, Torrance CA. All research was performed in accordance with relevant guidelines/regulations, and in accordance with the Declaration of Helsinki.

### Quantification of plasma TMAO

Serial plasma TMAO concentrations were measured using stored frozen (−80 ˚C) 12-h fasting blood samples collected at baseline (2000–2002; *n* = 6,796 participants) and exam 4 (2005–2007; *n* = 5633 participants) using established stable isotope dilution with on-line liquid chromatography tandem mass spectrometry (LC-MS/MS) methods^12^. Briefly, LC-MS/MS analyses were performed on a triple quadrupole mass spectrometer (LCMS-8050, Shimadzu Corporation, Kyoto, Japan) at the Cleveland Clinic Lerner Research Institute using d9(trimethyl)TMAO (d9-TMAO) as internal standards. TMAO and d9-TMAO were monitored using electrospray ionization in positive-ion mode with multiple reaction monitoring of parent and characteristic daughter ion transitions as previously described, including m/z 76.10 ◊59.10 and 76.10 ◊58.10 for TMAO, and 85.00◊ 66.15 and 85.00◊ 68.15 for d9-TMAO. At least three pooled quality control (QC) samples spanning between 0.5 μm and 200 μm TMAO levels were included with every batch (81 samples) analyzed. Assay performance metrics include laboratory intra-day and inter-day coefficient of variations for TMAO analyses that were < 6% across all QC samples; accuracy was > 97% for all pooled QC samples; Lower limit of detection = 0.02 μm, signal-to-noise ratio of 3 or greater; Lower limit of quantification = 0.05 μm, signal-to-noise ratio of 10 or greater; upper limit of quantification > 200 μm^12^.

We estimated time-varying TMAO exposure, with TMAO concentrations measured in 2000–2002 related to risk between 2000 and 2002 and 2005–2007, and the average of TMAO levels in 2000–2002 and 2005–2007, to risk from 2005 to 2007 through 2017. This specific approach for cumulative average has been widely used^13–15^ to reduce exposure misclassification due to within-subject variation in TMAO measures.

### Ascertainment of ASCVD

MESA event surveillance and classification have been described^16^. Participants were seen at in-person study visits every 2–3 years, with additional telephone interview contacts at intervals of 9–12 months, to inquire about interim hospital admissions, cardiovascular outpatient diagnoses, and deaths. Self-reported diagnoses were verified using medical records for hospitalizations and outpatient visits, death certificates, and next-of-kin interviews for out-of-hospital cardiovascular deaths^17^. Records were obtained on 98% of reported hospitalized cardiovascular events^18^ and 95% of reported outpatient diagnostic encounters. Trained personnel abstracted medical records, and two physicians independently classified the events and assigned incidence dates, with any differences resolved via discussion. The primary outcome of the present investigation was incident ASCVD, including the first definite or probable MI, CHD death, fatal or nonfatal stroke (not transient ischemic attack), resuscitated cardiac arrest, other atherosclerotic death, or other cardiovascular death. Additional details on MESA’s follow-up methods and event adjudication are at http://www.mesa-nhlbi.org.

### Covariates

At each study visit, standardized questionnaires collected data on age, sex, race/ethnicity, education, smoking, co-morbidities, and medications; physical examination was performed; and laboratory measurements were obtained. Smoking was categorized as never, former, or current; with current smoking further delineated as light (1–20 pack-years), moderate (20.1–40 pack-years), or heavy (> 40 pack-years). Habital dietary intake over the previous year was assessed at baseline using a 120-item self-administered food frequency questionnaire (FFQ) adapted to include Chinese cuisine^20^. Participants reported the frequency and usual serving size for each food item, based on which daily servings of fruits, vegetables, meat and alcohol were calculated^21^. Fasting glucose ≥ 126 mg/dL or hypoglycemic medication use defined diabetes mellitus. Resulting blood pressure (Dinamap model Pro 100) was assessed as the average of the second and third readings^22^. Height and weight were measured, and body mass index (BMI) calculated as weight (kg)/height (m) squared. Physical activity was ascertained using the MESA Typical Week Physical Activity Survey (TWPAS), identifying amounts of time spent in and frequency of various physical activities during a typical week in the prior month^11^. Minutes of activity were summed for each discrete type and multiplied by metabolic equivalent (MET) level. In our study, we used total intentional exercise. After an overnight fast, blood samples were collected to measure total cholesterol, high-density lipoprotein-cholesterol (HDL-C), and triglyceride levels; with LDL-C calculated using the Friedewald equation^23^. Using the colorimetric method and creatinine-based CKD-EPI (Chronic Kidney Disease Epidemiology Collaboration) equation, serum creatinine and estimated Glomerular Filtration Rate (eGFR) were calculated^24^. Family history of CVD was defined from self-reported history of myocardial infarction (MI) and stroke in first-degree relatives.

### Statistical analyses

We evaluated associations between plasma TMAO concentrations and ASCVD using Cox proportional hazards models with time-at-risk until the first event, other death, or censoring at the last follow-up (PROC PHREG procedure SAS)^25^. We tested the proportional hazards assumption by using extended Cox models with product terms of TMAO and covariates with time-dependent log of time to ASCVD event, and found no evidence of violation for TMAO or any covariate except prevalent diabetes at baseline. We addressed this by incorporating diabetes as a risk-set stratification variable^26^.

To assess association of long-term TMAO exposure, we used time-varying TMAO concentrations, with TMAO measured at baseline related to incident events through 2005–2007, and the average of baseline and 2005–2007 TMAO related to events occurring after 2005–2007. For participants with only one baseline TMAO measure (17%), that measured was carried forward. We evaluated TMAO continuously using the interquintile median range (IQR, defined as the difference between the median of the fifth and first quintiles) and in quintiles as indicator variables. We assessed the presence of nonlinear relationships using restricted cubic splines. To minimize potential confounding, we considered pre-specified covariates based on biologic interest, risk factors for ASCVD, or associations with TMAO or ASCVD. Covariates included baseline sex, race/ethnicity, enrollment site, education, and income, and time-varying age, self-reported health status, smoking status, physical activity, BMI, waist circumference, prevalent diabetes, alcohol intake, HDL-C, LDL-C, triglycerides, C-reactive protein, systolic and diastolic blood pressure, use of lipid-lowering medication, use of anti-hypertensive medication, use of antibiotics within the 2 weeks preceding TMAO measurement, and habitual diet including intakes of animal source foods (the sum of unprocessed red meat, processed meat, eggs, chicken, and fish), fruits, vegetables, and dietary fiber. We included both BMI and waist circumference in our models to minimize residual confounding by adiposity; and both education and income to minimize confounding by socioeconomic status. Time-varying covariates were updated from exam 4. We conducted analysis adjusting for dietary risk factors to determine whether the potential association with TMAO could be attributed to TMAO-containing food sources or other dietary factors correlated with healthy eating patterns and/or TMAO concentrations. In sensitivity analyses, we adjusted for eGFR^27^, which could be a mediator or confounder of the TMAO-ASCVD association given that TMAO is renally cleared and that experimental models show that TMAO damages the kidney, increases cystatin C, and reduces eGFR^28,29^. Missing covariate data (generally < 1%) were imputed using multiple demographic and risk variables using single multivariable imputation; in prior analyses, we have shown that such methods provide results very similar to multiple imputation^30^. We explored potential effect modification by baseline age, sex as a biological variable, race/ethnicity as a self-reported social construct, BMI, and eGFR (< 60 vs. ≥ 60 mL/min/1.73m2). Statistical significance of each multiplicative interaction term was assessed using the Wald test, with p-values for these exploratory analyses Bonferroni adjusted for multiple comparisons (α = 0.05/5 = 0.01). Analyses otherwise utilized a two-tailed alpha = 0.05 and were performed using Stata, release 14.0 (StataCorp), and SAS, version 9.4 (SAS Institute).

## Results

**Fig. 1:**
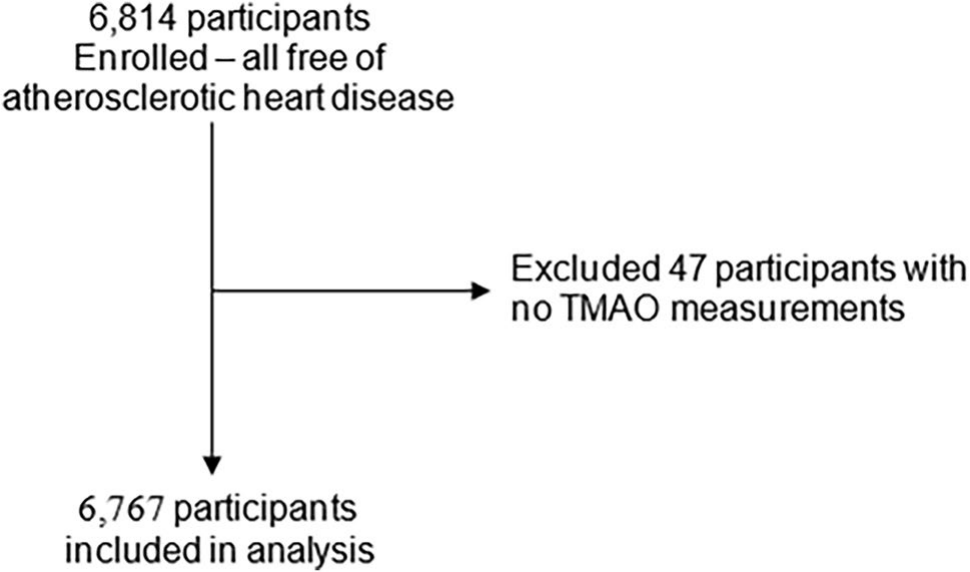
STROBE diagram for patient characteristics. Abbreviations: MESA = Multiethnic Study of Atherosclerosis; TMAO = trimethylamine *N*-oxide.

At baseline, mean (SD) age was 61.8 (10.1) years, and 38% of participants identified as White adults; 28%, Black; 22%, Hispanic; and 12%, Chinese (Table 1; Fig. 1). Mean plasma TMAO concentrations (mean ± SD µmol/L) were 5.2 ± 7.1 and 6.3 ± 9.2 at exams 1 and 4, respectively. The correlation between TMAO measures was moderate, with Spearman partial correlation coefficients adjusting for age, sex, and race-ethnicity, yielding a value of 0.35 (p-value < 0.001). Approximately 13% of participants had diabetes, 6% had low eGFR, 16% were taking lipid-lowering medications, 37% were on anti-hypertensive medications, and 3% had taken antibiotics in the previous 2 weeks. In crude (unadjusted) analyses across quintiles, those with higher circulating TMAO concentrations were more likely to be older and White; have diabetes, low eGFR, higher systolic blood pressure, and higher triglyceride levels; and be on lipid-lowering and anti-hypertensive medications.

**Table 1:** Baseline characteristics of 6,765 US adults free of cardiovascular disease at baseline according to plasma levels of trimethylamine N-oxide (TMAO) in the Multi-Ethnic study of atherosclerosis.

	Quintiles of TMAO				
	Q1	Q2	Q3	Q4	Q5
n	1,351	1,354	1,354	1,352	1,354
Median TMAO ((10th, 90th)), µmol/L	1.75 (1.09, 2.14)	2.61 (2.29, 2.95)	3.56 (3.14, 4.01)	4.98 (4.32, 5.96)	9.2 (6.67, 9.20)
**Demographics**					
Age, years	59 (10)	61 (10)	63 (10)	64 (10)	65 (10)
Female, n (%)	751 (55.6)	760 (56.1)	715 (52.8)	668 (49.4)	676 (49.9)
Race, n (%)					
White	399 (29.5)	523 (38.6)	532 (39.3)	579 (42.8)	574 (42.4)
Asian	216 (16)	157 (11.6)	151 (11.2)	121 (9)	156 (11.5)
Black	402 (29.8)	360 (26.6)	377 (27.8)	363 (26.9)	366 (27)
Hispanic	334 (24.7)	314 (23.2)	294 (21.7)	289 (21.4)	258 (19.1)
**Lifestyle and other risk factors**					
Income					
<$11,999	92 (6.8)	74 (5.5)	96 (7.1)	77 (5.7)	90 (6.6)
$12,000 to 24,999	226 (16.7)	225 (16.6)	266 (19.7)	237 (17.5)	237 (17.5)
$25,000 to $49,999	374 (27.7)	357 (26.4)	358 (26.4)	399 (29.5)	351 (25.9)
>$50,000	488 (36.1)	510 (37.7)	466 (34.4)	470(34.8)	469 (34.6)
≥ 12 years of education	966 (71.5)	971 (71.7)	963 (71.1)	969 (71.7)	929 (68.6)
Cigarette Smoking, n (%)					
Never	709 (52.5)	705 (52.1)	663 (49.0)	666 (49.3)	659 (48.7)
Former	436 (32.3)	475 (35.1)	522 (38.5)	521 (38.5)	526 (38.8)
Current	206 (15.2)	174 (12.8)	169 (12.5)	165 (12.2)	169 (12.5)
Physical activity), MET-min/week	1557 (2166)	1544 (2275)	1633 (2775)	1567 (2302)	1479 (2135)
Alcohol, drinks/week	2.4 (5)	2.8 (5.8)	2.6 (4.9)	2.7 (5.3)	2.6 (5.2)
BMI, kg/m2	27.6 (5.6)	28.2 (5.3)	28.3 (5.4)	28.9 (5.6)	28.6 (5.4)
Waist circumference, cm	95.2 (14.5)	97.5 (14)	98.5 (14.2)	100 (14)	99.5 (14.7)
Systolic blood pressure, mmHg	124.8 (21.5)	124.8 (20.8)	126.6 (20.7)	128.3 (21.6)	128.4 (22.5)
Diastolic blood pressure, mmHg	72.4 (10.2)	71.6 (10.2)	71.9 (9.8)	72.1 (10.7)	71.6 (10.4)
**Biochemical**					
HDL cholesterol, mg/dL	51.7 (14.4)	52 (15.8)	50.7 (14.4)	50.4 (14.8)	50.1 (14.7)
LDL cholesterol, mg/dL	117.9 (31.7)	118 (31.7)	118.2 (32.6)	114.7 (31.5)	115.4 (32.9)
Triglycerides, mg/dL	127 (87.8)	130.3 (96.8)	133.5 (87.6)	134.3 (82.1)	132.8 (89.1)
Estimated glomerular filtration rate, mL/min/1.73m2	98.2 (14.8)	93.4 (14.9)	90.3 (16.6)	86.2 (18)	82.8 (21.7)
**Medical history**, n (%)					
Prevalent diabetes	163 (12.1)	159 (11.7)	209 (15.4)	212 (15.7)	191 (14.1)
Lipid lowering medication	188 (13.9)	159 (11.7)	218 (16.1)	245 (18.1)	244 (18)
Anti-hypertensive medication	364 (26.9)	434 (32.1)	507 (37.4)	590 (43.6)	621 (45.9)
Antibiotics use in past 2 weeks	50 (3.7)	33 (2.4)	38 (2.8)	41 (3)	42 (3.1)
eGFR, n (% <60)	6 (0.4)	24 (1.8)	55 (4.1)	96 (7.1)	196 (14.5)
**Dietary habits**					
Fruits, servings/d	1.9 (1.8)	1.9 (1.6)	1.9 (1.6)	2 (1.7)	1.9 (1.5)
Vegetables, servings/d	1.9 (1.3)	1.8 (1.3)	1.9 (1.4)	1.8 (1.4)	1.9 (1.4)
Fiber, g/d	18.8 (10.4)	18.7 (10.4)	18.9 (10.7)	18.9 (10.4)	18.2 (9.3)
Unprocessed and processed red meat, servings/d	0.4 (0.6)	0.5 (0.6)	0.5 (0.6)	0.5 (0.6)	0.5 (0.5)
Poultry, servings/d	0.3 (0.4)	0.3 (0.4)	0.3 (0.3)	0.3 (0.3)	0.3 (0.3)
Eggs, servings/d	0.1 (0.2)	0.1 (0.2)	0.1 (0.3)	0.1 (0.3)	0.1 (0.3)
Fish, servings/d	0.3 (0.4)	0.2 (0.3)	0.2 (0.3)	0.2 (0.3)	0.2 (0.3)

Values are mean (SD) for continuous variables and N (%) for categorical variables.

TMAO, trimethylamine-N-oxide, MET-min/week, metabolic equivalents per minute/week, eGFR, estimated glomerular filtration rate.

**Fig. 2:**
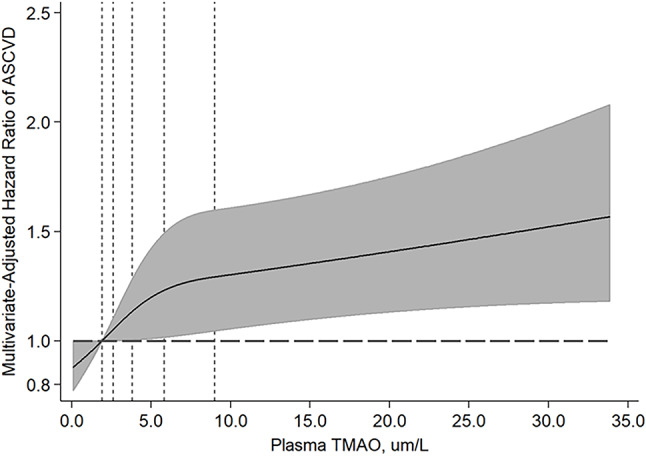
Multivariate-adjusted relationships between plasma TMAO and ASCVD evaluated using restricted cubic splines. Knots were evaluated at the 10th, 50th, and 90th percentiles. Dotted vertical lines represent, from left to right, the 10th, 25th, 50th, 75th, and 90th percentiles of circulating TMAO. Covariates are specified in Table 2. The top 1% of the exposure distribution was omitted for better visualization.

**Fig. 3:**
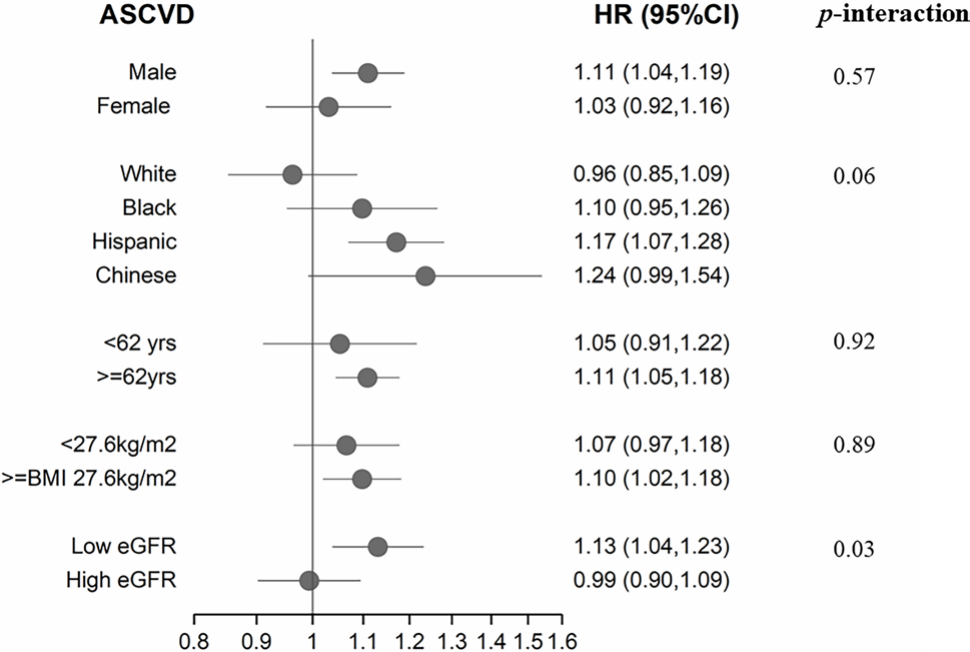
Multivariate-adjusted relationships between plasma TMAO and ASCVD in key subgroups. Hazard ratios (and 95% confidence intervals) are shown for continuous analyses comparing the midpoint of the highest vs. lowest quintile of plasma TMAO among 6,765 US adults in the Multi-Ethnic Study of Atherosclerosis, stratified by sex, race/ethnicity, age, body mass index (BMI), and renal function (estimated glomerular filtration rate below or above 60 mL/min/1.73 m^2^).

During a median follow up of 11.1 years (2000–2017), 852 ASCVD events occurred. In multivariate models adjusting for sociodemographics, lifestyle, metabolic risk factors, blood lipids, and medication use, higher plasma TMAO concentrations were associated with 9% higher risk of ASCVD in continuous analyses (per IQR, HR: 1.09, 95%CI: 1.03–1.15) and 32% higher risk across highest quintile (HR: 1.32, 95%CI: 1.01–1.73; p-trend = 0.02) (Table 2). This association remained unchanged with further adjustments for dietary risk factors. There was no significant evidence of nonlinear relationships between plasma TMAO and ASCVD risk based on restricted cubic splines (p non-linearity = 0.09) (Fig. 2). Overall, the findings were consistent when evaluating stroke and myocardial infarction (both fatal and non-fatal), with an 11% higher risk of stroke observed in continuous analyses (per IQR increase, HR: 1.11; 95% CI: 1.02–1.22), and a 12% higher risk of myocardial infarction (HR: 1.12; 95% CI: 1.04–1.21).Our exploratory subgroup analyses showed no statistically significant differences in the TMAO-ASCVD association across subgroups of age, sex, BMI, or eGFR (< 60 vs. ≥ 60 mL/min/1.73 m²), with P-interaction values > 0.01 each. Although effect modification by race/ethnicity was not statistically significant, the central effect estimate appeared possibly larger among Hispanic adults (HR: 1.17, 95% CI: 1.07, 1.28) and Chinese adults (HR: 1.24, 95% CI: 0.99, 1.54). The TMAO-ASCVD association also appeared stronger in individuals with a baseline eGFR < 60 mL/min/1.73 m² (Table 3; Fig. 3).

**Table 2:** Association of long-term plasma TMAO measures with incident atherosclerotic cardiovascular disease (ASCVD)among 6,767 US Adults in the Multi-Ethnic Study of Atherosclerosis.

	Q1	Q2	Q3	Q4	Q5	*P*-trend	Continuous, per -IQR^a^
Median TMAO, µmol/L	1.75	2.61	3.56	4.98	9.2		
Total number of participants/ASCVD cases	1351/73	1354/112	1354/180	1352/231	1354/256		
Person-years of follow-up	18,445	18,429	18,014	17,459	17,128		
Hazard ratios (95% CI)^b^							
Demographic-adjusted	REF	1.06 (0.79, 1.42)	1.25 (0.95, 1.64)	1.36 (1.04, 1.78)	1.51 (1.15, 1.97)	0.0003	1.11 (1.05, 1.17)
Multivariable-adjusted	REF	1.02 (0.76, 1.37)	1.17 (0.89, 1.54)	1.24 (0.94, 1.62)	1.32 (1.01, 1.73)	0.02	1.09 (1.03, 1.15)
Multivariable plus diet	REF	1.02 (0.75, 1.37)	1.17 (0.88, 1.54)	1.23 (0.94, 1.62)	1.33 (1.01, 1.74)	0.01	1.09 (1.03, 1.15)

Long-term TMAO levels were assessed by using serial measures, with TMAO concentrations in 2000-2002 related to risk between 2000-2002 and 2005-2007; and the average of TMAO levels in 2000-2002 and 2005-2007 related to risk from 2005-2007 through 2017. ASCVD included MI, resuscitated cardiac arrest, fatal and non-fatal stroke, CHD death, other atherosclerotic death, and other CVD death.

^a^The difference between the midpoint of the top and bottom quintiles (interquintile range, IQR)=7.5 µmol/L.

^b^The demographic adjusted model includes age (years), sex (male, female), race/ethnicity (White, Black, Black, Hispanic, Chinese) and field center. The multivariable-adjusted model further includes education (<high school, high school, some college, college graduate), income (<$11,999, $12,000-$24,999, $25,000-$49,999, >$50,000/y), as well as time-varying pack-years of cigarette smoking, alcohol intake (drinks per week), physical activity (active and inactive leisure, MET-min/week), waist circumference (cm), lipid lowering medication (yes/no), anti-hypertensive medication (yes/no), antibiotics (yes/no), prevalent diabetes (yes/no), high-density lipoprotein cholesterol (mg/dL), low-density lipoprotein cholesterol (mg/dL), triglycerides (mg/dL), systolic blood pressure (mmHg), diastolic blood pressure (mmHg). The multivariable plus diet model includes additional adjustment for intakes of fruits (servings/week), vegetables (servings/week), fiber (g/d), and red meat (servings/week).

### Sensitivity analysis

After further adjustment for eGFR, which could be a potential mediator or confounder, the association was attenuated and no longer statistically significant: HR for extreme quintiles: 1.15, (95%CI: 0.87–1.53; p-trend = 0.28; continuous HR: 1.05 (95%CI: 0.99–1.12)). Associations between TMAO and ASCVD were not appreciable altered after excluding 1,145 participants with only one TMAO measure (multivariable-adjusted HR (95% CI): 1.09 (1.01–1.17) per IQR and 1.35 (0.98–1.86); p-trend < 0.01 for extreme quintiles of TMAO concentrations. In addition, associations remained unchanged in sensitivity analysis excluding 236 individuals who developed CVD within two years of the baseline (multivariable-adjusted HR (95% CI) per IQR 1.10 (1.04–1.17), and across quintiles 1.42, 95%CI: 1.06–1.91; p-trend < 0.01).

**Table 3:** Association of long-term plasma TMAO levels with ASCVD, according to race/ethnicity.

	Hazard ratio (95% CI) per IQR^a^
All (n=6,765, 852 cases)	1.09 (1.03, 1.15)
Whites (n=2,607; 328 cases)	0.96 (0.85, 1.09)
Blacks (n=1,868; 246 cases)	1.10 (0.95, 1.26)
Hispanics (n=1,489; 200 cases)	1.17 (1.07, 1.28)
Asians (n=801; 78 cases)	1.24 (0.99, 1.54)

Long-term TMAO levels were assessed by using serial measures, with TMAO concentrations in 2000-2002 related to risk between 2000-2002 and 2005-2007; and the average of TMAO levels in 2000-2002 and 2005-2007 related to risk from 2005-2007 through 2017. ASCVD included MI, resuscitated cardiac arrest, fatal and non-fatal stroke, CHD death, other atherosclerotic death, and other CVD death.

^a^The difference between the midpoint of the top and bottom quintiles (interquintile range, IQR)=7.5 µmol/L. Associations adjusted for age (years), sex (male, female), race/ethnicity (White, Black, Black, Hispanic, Chinese; when applicable) field center, education (<high school, high school, some college, college graduate), income (<$11,999, $12,000-$24,999, $25,000-$49,999, >$50,000/y), as well as time-varying pack-years of cigarette smoking, alcohol intake (drinks per week), physical activity (active and inactive leisure, MET-min/week), waist circumference (cm), lipid lowering medication (yes/no), anti-hypertensive medication (yes/no), antibiotics (yes/no), prevalent diabetes (yes/no), high-density lipoprotein cholesterol (mg/dL), low-density lipoprotein cholesterol (mg/dL), triglycerides (mg/dL), systolic blood pressure (mmHg), diastolic blood pressure (mmHg).

## Discussion

In this large, prospective, community-based cohort of multi-ethnic/racial backgrounds and with serial TMAO measures over time, higher plasma TMAO was associated with 32% higher risk of incident ASCVD comparing the highest quintile to the lowest quintile. This association was present after adjustment for sociodemographics, lifestyle habits, metabolic risk factors, blood lipids, and medication use. This included adjustment for dietary intakes of fruits, vegetables, and red meat, suggesting that the TMAO-ASCVD relationship extends beyond these factors. Importantly, the association with ASCVD remained significant after adjusting for smoking, diabetes, adiposity, blood pressure, and lipids, suggesting mechanisms linking TMAO and atherosclerotic risk beyond traditional risk factors.

A number of experimental studies support the biologic plausibility and potential causality of our observed associations. TMAO may increase atherosclerosis and plaque vulnerability through effects on inflammation^31^, oxidative stress, endothelial dysfunction, and thrombosis^32^. TMAO may also worsen glycemic control^33^ and promote metabolic dysfunction^34^, which may increase the risk of microvascular disease^35^ and macrovascular ASCVD. At the cellular level, TMAO promotes foam cell formation in artery walls by increasing expression of pro-atherogenic scavenger receptors^36–38^, reducing reverse cholesterol efflux^2^, and enhancing platelet reactivity through increased Ca^2+^ release from intracellular stores^39^. TMAO may also directly impact vascular endothelial cell Tissue Factor expression to heighten thrombotic potential^25^. Each of these multiple pathways could influence the development and incidence of ASCVD.

The present study represents a unique, community-based multi-ethnic population who were free of clinical ASCVD at baseline. Our finding of a positive association between TMAO and ASCVD in this community-based study supports and greatly extends the results of prior observational studies, many of which were in clinical samples of patients with preexisting disease^43^, –^44^) particularly since we evaluated a racially and ethnically diverse population and were also able to adjust for a wide variety comorbidities, risk factors, and social/demographic factors typically not available as covariates in most other studies (e.g. income, education, physical activity, and dietary habits). We also leveraged serial measures of TMAO, reducing misclassification due to changes in its levels over time. To our knowledge, only one prior study has reported on associations of serially measured TMAO with ASCVD, in the Cardiovascular Health Study (CHS) comprising adults age 65 + years at baseline^45^. In that study, higher long-term levels of plasma TMAO associated with higher risk of both incident ASCVD and recurrent ASCVD. In CHS, the highest quintile HR for incident CVD was 1.21 (95% CI: 1.02, 1.42), similar to our current results in MESA (HR: 1.33, 95% CI :1.02, 1.74).

In exploratory analyses by race and ethnicity, the association between TMAO and ASCVD was numerically larger among Hispanic and Asian participants as compared to White participants, but these differences between races/ethnicities were not statistically significant. To our knowledge, this is the first assessment of TMAO and incident ASCVD in a diverse multi-racial/ethnic cohort. Whether the potentially larger effects among Hispanic and Asian participants are due to chance, confounding, or biology needs further exploration in other cohorts and experimental studies. In a recent systematic review of ASCVD and TMAO, few studies evaluated TMAO and ASCVD in non-Caucasian populations, and these were generally small and limited to clinical cohorts with prevalent major clinical diseases^49^. –^50^)

In the current study, the TMAO-ASCVD association was attenuated after adjustment for eGFR; and also appeared possibly stronger in those with mildly reduced renal function at baseline (eGFR < 60). Because TMAO causally reduces eGFR, this could be a mediator in the causal pathway of its effects. Similar findings were seen in our prior work among older adults in CHS^13,28,40–45^, although only among individuals without prevalent ASCVD at baseline. Well established mechanistic evidence shows that TMAO can directly impair renal function. Feeding mice with choline or TMAO reduces GFR, raises plasma cystatin C and urine albumin levels, and increases tubulointerstitial fibrosis^28,29^. Moreover, these renal impairments can be prevented by targeted inhibition of gut microbial TMAO generation^29,46^. TMAO also reduces albumin uptake by human proximal tubular cells and suppresses expression of megalin, an albumin-binding surface receptor on tubular epithelial cells critical for urine protein reabsorption^48^. In line with these experimental findings, higher plasma TMAO levels associate with higher risk of incident CKD and decline in kidney function in community-based cohorts^15^. Our new findings support the need for further investigation of this interplay between diet, microbiome-related metabolites, renal decline, and ASCVD.

Our investigation has several strengths. The multi-racial and ethnic cohort across ages 45–84 years at baseline increases generalizability, and low loss to follow-up reduces potential for bias. The prospective design among individuals free of ASCVD allows assessment of temporality and minimizes reverse causation, while the population-based cohort design minimizes selection bias. The large sample size and extended follow-up provided a large number of events and statistical power to detect relevant associations. Information on a wide array of ASCVD risk measured using standardized methods^11^, facilitating multivariable adjustment to reduce confounding. ASCVD outcomes were identified and confirmed by a comprehensive review and centralized adjudication process, reducing the potential for missed or misclassified outcomes. The use of serial measures of TMAO with time-varying estimates reduced exposure misclassification from variations in TMAO concentrations over time. The time-varying Cox regression models appropriately allow adjustment for exposures and covariate values, including age, also increases the stability of the measure and thus the generalizability of the results, and potential clinical utility of the findings.

Potential limitations should be considered. Due to the observational nature of our study, residual confounding by unknown or unmeasured factors may be present. However, our results were robust to adjustment for multiple cardiovascular risk factors as well as sociodemographics and dietary and other lifestyle habits. Data on factors associated with TMAO production, such as composition of gut microbiota and hepatic flavin-containing monooxygenase 3 (FMO3) activity, were not collected, which should be considered in future studies. As TMAO levels were measured prospectively, before development of disease, any remaining within-individual variation is likely to attenuate findings toward the null, reducing the strength of observed associations. The population was U.S.-based; hence findings are not necessarily generalizable to other nations.

Several lines of thought suggest that TMAO accelerates atherosclerosis by reducing reverse cholesterol transport and promoting vascular inflammation, which can lead to lipid accumulation and progression of atherosclerosis. This is concordant with several recent meta-analysis that demonstrate an independent relationship between all-cause mortality, CVD mortality, major adverse cardiovascular events, hypertension, DM, and glomerular filtration rate^51,52^.

These new findings support the role of the diet-microbiome axis for atherosclerotic risk in multi-ethnic US populations, indicating a need to test dietary and pharmacologic interventions in individuals with elevated gut microbial metabolites like TMAO as a potential effective strategy in mitigating future ASCVD.

## Electronic supplementary material

Below is the link to the electronic supplementary material.

Supplementary Material 1

Supplementary Material 2

## Data Availability

The research data is available at - https://internal.mesa-nhlbi.org/internal/data/datasets-and-documentation/events.

## Abbreviations

ASCVD: Atherosclerotic cardiovascular disease
BMI: Body mass index
CHS: Cardiovascular health study
CKD-EPI: Chronic kidney disease epidemiology collaboration
eGFR: Estimated Glomerular Filtration Rate
FMO3: Flavin-containing monooxygenase 3
HDL-C: High-density lipoprotein-cholesterol
IQR: Interquintile median range
MET: Metabolic equivalent
MI: Myocardial infarction
TMAO: Trimethylamine N-oxide
TWPAS: Typical week physical activity survey
