# Supplementary material for: Trimethylamine-N-oxide (TMAO) and risk of incident cardiovascular events in the multi ethnic study of Atherosclerosis
Source: Sci Rep. 2025 Jul 2;15:23362. doi: 10.1038/s41598-025-05903-3 (PMC12222875; doi:10.1038/s41598-025-05903-3)
Supplement: Supplementary file 1 — Supplementary Material 1 [file 41598_2025_5903_MOESM1_ESM.docx]

SUPPLEMENT -

**Trimethylamine-*N*-oxide (TMAO) and Risk of Incident Cardiovascular Events in the Multi Ethnic Study of Atherosclerosis**

Budoff et al.

Supplemental Table 1: Correlations between intakes of animal food sources and time-varying concentration of plasma TMAO among 6,767 US Adults in the Multi-Ethnic Study of Atherosclerosis^1^

|  | Red meat | Poultry | Eggs | Fish |
| --- | --- | --- | --- | --- |
| TMAO | 0.06 | -0.04 | 0.03 | 0.05 |

**^1^**Estimated using partial Spearman correlation analysis adjusting for age, sex, race. P-values for all coefficients was below 0.01.

Time-varying TMAO exposure was assessed by using time varying measures, i.e., TMAO concentrations in 2000-2002 were related to risk between 2000-2002 and 2005-2007; the average of TMAO levels in 2000-2002 and 2005-2007, to risk from 2005-2007 through 2017

TMAO, Trimethylamine N-oxide
