# Supplementary material for: Trimethylamine-N-oxide (TMAO) and risk of incident cardiovascular events in the multi ethnic study of Atherosclerosis
Source: Sci Rep. 2025 Jul 2;15:23362. doi: 10.1038/s41598-025-05903-3 (PMC12222875; doi:10.1038/s41598-025-05903-3)
Supplement: Supplementary file 2 — Supplementary Material 2 [file 41598_2025_5903_MOESM2_ESM.docx]

| **Supplemental Table – Association of long-term plasma TMAO measures with incident stroke and Myocardial infarction among** **6,767 US Adults in the Multi-Ethnic Study of Atherosclerosis.^1^** |
| --- |
| \|  \| Q1 \| Q2 \| Q3 \| Q4 \| Q5 \| *P*-trend \| Continuous, per -IQR^2^ \| \| --- \| --- \| --- \| --- \| --- \| --- \| --- \| --- \| \|  \|  \|  \|  \|  \|  \|  \|  \| \| Median TMAO, µmol/L \| 1.75 \| 2.61 \| 3.56 \| 4.98 \| 9.2 \|  \|  \| \| Stroke (n=307) \| REF \| 1.04 (0.64, 1.67) \| 0.85 (0.53, 1.36) \| 1.13 (0.77, 1.75) \| 1.36 (0.88, 2.11) \| 0.02 \| 1.11 (1.02, 1.22) \| \|  \|  \|  \|  \|  \|  \|  \|  \| \| Myocardial infarction (n=336) \| REF \| 1.08 (0.68, 1.73) \| 1.27 (0.82, 1.97) \| 1.25 (0.81, 1.92) \| 1.48 (0.96, 2.27) \| 0.048 \| 1.12 (1.04, 1.21) \| \|  \|  \|  \|  \|  \|  \|  \|  \|   Long-term TMAO levels were assessed by using serial measures, with TMAO concentrations in 2000-2002 related to risk between 2000-2002 and 2005-2007; and the average of TMAO levels in 2000-2002 and 2005-2007 related to risk from 2005-2007 through 2017. ASCVD included MI, resuscitated cardiac arrest, fatal and non-fatal stroke, CHD death, other atherosclerotic death, and other CVD death. |
| The difference between the midpoint of the top and bottom quintiles (interquintile range, IQR)=7.5 µmol/L.  Multivariable-adjusted model includes age (years), sex (male, female), race/ethnicity (White, Black, Black, Hispanic, Chinese) and field center, education (<high school, high school, some college, college graduate), income (<$11,999, $12,000-$24,999, $25,000-$49,999, >$50,000/y), as well as time-varying pack-years of cigarette smoking, alcohol intake (drinks per week), physical activity (active and inactive leisure, MET-min/week), waist circumference (cm), lipid lowering medication (yes/no), anti-hypertensive medication (yes/no), antibiotics (yes/no), prevalent diabetes (yes/no), high-density lipoprotein cholesterol (mg/dL), low-density lipoprotein cholesterol (mg/dL), triglycerides (mg/dL), systolic blood pressure (mmHg), diastolic blood pressure (mmHg). |
